# Cognitive Stimulation in Older Adults: An Innovative Good Practice Supporting Successful Aging and Self-Care

**Published:** 2019-01-06

**Authors:** J Apóstolo, E Bobrowicz-Campos, I Gil, R Silva, P Costa, F Couto, D Cardoso, A Barata, M Almeida

**Affiliations:** 1 Nursing School of Coimbra, Coimbra; 2 The Health Sciences Research Unit: Nursing, Nursing School of Coimbra, Coimbra; 3 Universidade Católica Portuguesa, School of Nursing, Porto

**Keywords:** older adults, cognitive frailty, cognitive impairment, cognitive stimulation, reminiscence

## Abstract

The project Cognitive Stimulation in Older Adults: Intervention on Cognitive Frailty and Promotion of Self-Care (in brief the ECOG project) arises in a context of worldwide demographic aging, and is fostered by the need to provide a sustainable solution to the progressive increase in the prevalence of age-related cognitive impairment. The main goal of the ECOG project is to promote active citizenship in old age through the empowerment for autonomy and self-care. Namely, the ECOG team is working on the development of evidence-based programs and tools that promote gains in health in cognitively frail and cognitively impaired older adults from the community. It is also working on the transfer of ECOG products to the practice of health and social care, promoting active involvement of geriatric care institutions in the implementation of the ECOG programs and tools, and ensuring appropriate training of professionals. Finally, the ECOG team is deploying a digital platform to reach out to the broadest audience possible and support the remote access and scaling up of the ECOG products.

The impacts expected at an individual level include improvements in cognition, functionality, and autonomy of older adults, with simultaneous reduction of depressive symptomatology, and increase in quality of life of both person cared and his/her caregiver. Regarding societal gains, we anticipate an increase in life expectancy and significant postponement of institutionalization associated with geriatric problems. We also believe that the wide implementation of the ECOG products will reduce the costs of interventions for cognitively impaired citizens, contributing to sustainability and efficiency of health systems.

## I. INTRODUCTION

Portugal is one of the European countries most affected by the phenomenon of demographic aging [1], with the aging and dependency rates for the older persons reaching in 2015 the values of 143.9% and 31.4%, respectively. Demographic changes have a dramatic impact on social and family structures due to age-related worsening health status and functionality, and the subsequent need to activate mechanisms that mitigate their effects. The lack of support measures for informal caregivers makes institutionalization one of the most used social responses, especially in cases where older persons are cognitively and/or functionally impaired, and when they cannot be adequately accompanied in activities of daily living [2].

The international entities working to improve health and well-being throughout the life cycle, such as the World Health Organization (WHO), have recognized the phenomenon of demographic aging as one of the major social and economic challenges of the XXI century [3]. As such, the European Commission has identified the improvement in the sustainability of healthcare systems as one of the priority goals under the Program Horizon 2020, and the European Innovation Partnership on Active and Healthy Ageing (EIP on AHA) has launched tools to support the large-scale adoption of good practices with a focus on successful aging [4][5]. With this change in political perspective, an opportunity window for re-adaptation of health and social care practice was opened, and conditions for the gradual implementation of new health paradigm were created.

One of the good practices identified in the EIP on AHA framework was *Cognitive Stimulation and Brain Fitness* developed in Coimbra, Portugal, with the support of the Health Sciences Research Unit: Nursing of the Nursing School of Coimbra [5]. The main objective of this practice was to provide a group-based cognitive stimulation program to community-dwelling and institutionalized older adults and to assess its impact on health-related outcomes. Within this practice, the programs *Making a Difference I* [6] and *Making a Difference II* [7] were culturally adapted and validated [8][9], and then implemented in the aged population from the Central Region of Portugal. To increase the impact of the *Cognitive Stimulation and Brain Fitness* practice, it became necessary to create mechanisms that would support its widespread dissemination and replication. That’s how the ECOG project comes about.

The ECOG project, which stands for *Cognitive stimulation in older adults: intervention on cognitive frailty and promotion of self-care*, aims to increase autonomy and self-care in older adults by supporting the establishment and diffusion of stakeholder-centered strategies that empower and encourage communities for active involvement in promoting successful aging. The ECOG project was built in line with the Strategic Implementation Plan of the EIP on AHA [4], especially with the priority areas set out for the Action Group A3 (Lifespan health promotion and prevention of age-related frailty and disease), focusing its efforts on the creation of mechanisms that assist active ageing and independent living, and which prevent loss of functionality and autonomy. The ECOG project is also aligned with national policies on health, education and science, including the National Program for the Health of the Older Adults [10] and the Strategic Plan for the Therapeutic Approach to Cognitive Changes [11], both published by the Portuguese Directorate-General for Health, and the National Strategy for Research and Innovation for Intelligent Specialization: Health - Aging and Active Life [12]. The ECOG project is funded by the Nursing School of Coimbra, with the Health Sciences Research Unit: Nursing contributing to its development.

### Objectives of the ECOG project

The main goal of the ECOG project is to promote active citizenship in old age through empowerment for autonomy and self-care. To achieve this goal, three organizing axes have been identified, including evidence synthesis, the establishment of good practice and transfer of good practice to different healthcare contexts. The specific objectives defined within the three axes are as follows:

1. To consolidate the best available evidence on non-pharmacological interventions designed to prevent the progression of cognitive decline.
2. To elaborate interventional programs that prevent cognitive decline in older adults and enhance their quality of life and well-being.
3. To validate interventional programs with regard to feasibility, appropriateness, meaningfulness, and effectiveness.
4. To culturally adapt and validate instruments to support comprehensive geriatric assessment.
5. To transfer the ECOG products to geriatric care.
6. To provide educational materials and training for the autonomous implementation of the ECOG programs.
7. To deploy a technological platform to support remote access to the ECOG products and scaling up the good practice.
8. To replicate a good practice in new healthcare contexts.

## II. METHODOLOGY

The methodology of the ECOG project combines different approaches. The process of evidence synthesis (objective 1) was based on the methodological procedures for systematic reviews of effectiveness research, defined by the Joanna Briggs Institute (JBI) [13]. In total, four systematic reviews were conducted. Two of them focused on the effectiveness of reminiscence-based therapy on cognition, depressive symptoms and quality of life in institutionalized older adults [14] and in community-dwelling older adults [15]. One systematic review synthesized the evidence on the effectiveness of multisensory stimulation in managing neuropsychiatric symptoms in older adults with the major neurocognitive disorder [16]. Another one examined the effectiveness of caregiver-provided cognitive interventions on cognition, social functioning and quality of life among older adults with the major neurocognitive disorder [17].

The establishment of good practice (objectives 2, 3 and 4) occurred in several stages. Initially, two intervention programs to prevent progression of cognitive impairment in older adults were elaborated (objective 2). The first, a Reminiscence-Based Therapy provided in a group, was entirely designed by the ECOG team [18]. The second, a Cognitive Stimulation Program provided individually to older adults with dementia by their caregivers, was developed based on the structured therapy *Making a Difference 3* [19]. The process of cultural adaptation and validation of both programs followed the guidelines of the Medical Research Council for complex interventions [20]. For the adaptation of the Cognitive Stimulation Program, the Formative Method for Adapting Psychotherapy was additionally considered [21]. Citizens’ involvement was promoted throughout the different phases of the process, from the preliminary development, through modeling and field testing, to the consensus conference on the final products.

Posteriorly, several pilot studies of randomized controlled or quasi-experimental design were conducted (objective 3) to verify whether the intervention programs developed under the ECOG project were feasible, appropriate, meaningful and effective, as proposed in the JBI model of healthcare evidence generation [22]. Simultaneously, the process of cultural adaptation and validation of instruments that assess different domains of functioning in old age was carried out (objective 4). The instruments considered in this process were the 6-item Cognitive Impairment Test [23] and the Quality of Carer-Patient Relationship Scale [24]. The brief version of the World Health Organization Quality of Life – Module for Older Adults [25][26] was also developed.

Regarding the transfer to clinical practice (objectives 5, 6, 7 and 8), the products resulting from the ECOG project and its precedents were disseminated in the community, day centres, and nursing homes. Initially, only the Central Region of Portugal was involved. Posteriorly, the implementation of this good practice was extended to the North Region, Alentejo, and Ribatejo (objective 5). To support the dissemination process, the ECOG products have been designed in an attractive and user-friendly way (objective 6), and a technological platform enabling broader access to these products has been deployed (objective 7). Simultaneously, health and social care professionals working in the partner institutions were trained in the autonomous implementation of the intervention programs, in order to guarantee their future replication (objective 6). New contexts that could potentially benefit from the expansion of the good practice established by the ECOG team (objective 8) were also identified.

## III. EXPECTED OUTCOMES

The ECOG project will offer products and strategies to foster dissemination and replication of the innovative good practice in the community, widely contributing to the establishment of an integrated and personalized approach to geriatric healthcare. By encouraging the synergistic work between different stakeholders, the ECOG team will create opportunities for the exchange of knowledge and practices on successful aging and for the creation of strategies that allow maximization of older persons’ functionality for as long as possible. So far, the implementation of the ECOG programs involved more than 2000 seniors and dozens of institutions. The positive effects observed at an individual level included improvement in cognition, functionality, autonomy, and quality of life, with simultaneous reduction of depressive symptomatology [27][28]. However, the latter finding was not consistent. The impacts observed at an institutional level included (i) improvements in communication between the seniors and professionals, (ii) empowerment of professionals in the use of strategies that combine societal, community and clinical contexts and that address the whole life cycle, and (iii) capacitation of the professionals to encourage and empower older adults and their caregivers to take responsibility for their well-being.

The ECOG project will also provide recommendations to support the implementation of intervention programs. For this purpose, the experiences and perceptions of end-users and intervention providers are being mapped, with a special focus on factors that may interfere with the successful implementation of the ECOG programs. As an example, the preliminary results, obtained from the ECOG project partner institutions, have shown that formal training on program implementation and the clear articulation of all professionals about the schedule of activities and use of physical spaces are crucial to increase the impact of intervention programs. The need for better articulation with seniors to guarantee their commitment to the program was also identified. The inclusion of the recommendations in educational materials produced within the project’s framework will allow the replication of the programs’ success across a range of contexts.

The educational materials produced by the ECOG team will be made available to the citizens through a technological platform created specifically for this purpose. The use of the platform will foster the exchange of knowledge between different stakeholders working in the field of geriatric care. It will also amplify the use of ECOG products, increasing the potential range of the associated benefits. We expect that scaling up of such good practice will contribute to the increase in life expectancy and to avoidance/postponement of unnecessary / too early institutionalization of older adults.

## IV. DISCUSSION

The ECOG project presents multiple advantages at individual and institutional level. The implementation of group and home-based intervention programs allows the creation of environments where seniors learn and have fun and where their experience of social inclusion is strengthened. Active interaction with others contributes to the maximization of social and cognitive skills [27][28], which, in turn, positively impacts the individual’s adherence and commitment to ongoing or new programs, improving his/her health status and quality of life [27]. Other health gains include the reduction of isolation, loneliness, agitation and depressive symptoms [16], as well as the enhancement of functionality and autonomy [28]. All these gains can positively influence the dynamics of the family system and improve communication among its members. They can also indirectly affect the quality of life of caregivers.

Some of the ECOG products were designed to be used in a home-based setting. The availability of the intervention programs (in terms of structure, contents and implementation principles) can be seen as a factor that promotes the caregiver’s active involvement in the process, with the benefits being reflected in a significant reduction in the caregiver’s workload, improvement in the relationship with the cared person, and overall increase of caregiver’s well-being. Other expected benefits include the increase of caregivers’ health literacy and the adoption of healthy behaviours.

The advantages of professionals involved in the ECOG actions are related with fostering the transfer of scientific knowledge to daily clinical practice. They also include empowerment in the use of more individualized and person-centered approach to care, and improvement of the practical knowledge about the tools that support comprehensive geriatric assessment.

At an institutional level, the advantages include the possibility of using programs that are structured, easy-to-apply and highly accepted by seniors. The cost-benefit ratio of these programs is good since they do not require highly specialized professionals or specific material resources. In addition, the ECOG intervention programs show benefits in health that are comparable [29] or even higher [30] to those resulting from pharmacological interventions.

The Nursing School of Coimbra is responsible for adapting this good practice to the different contexts of geriatric care and for its subsequent validation, promoting the use of ECOG products by new partners. Although there are formal partnership protocols established between the Nursing School of Coimbra and other partner institutions and organizations, the latter have the autonomy to manage the intervention processes according to their needs and available resources. This autonomy is highly relevant, since it allows better management of factors related to the success of the ECOG programs, increasing their sustainability and facilitating their future replication. However, the additional mechanisms that support community outreach need to be identified, in order to multiply individual and societal gains and reduce overall costs of interventions for older citizens with cognitive impairment.

## V. CONCLUSION

The good practice established under the ECOG project is an innovative approach that supports successful aging. It focuses on the needs of older adults with cognitive impairment, their formal and informal caregivers, and institutions working in the field of geriatric care, and presents multiple advantages over other existing programs. As this practice has a potential to reduce the costs of interventions for cognitively impaired older citizens, contributing to sustainability and efficiency of health systems, efforts are made to integrate it into the health and social care provided to the aged population. However, it is still necessary to explore new research perspectives in the field of geriatric care, in order to reinforce the transfer of evidence to clinical practice and to enable impactful changes in its use.
